# Metagenomic next-generation sequencing (mNGS) versus tissue culture technique (TCT) in diagnosis of spinal infection: a systematic review and meta-analysis

**DOI:** 10.1038/s41598-025-06759-3

**Published:** 2025-07-01

**Authors:** Yan-Cheng Li, Yan-Xiao Liu, Lei Li, Hua Wang, Zi-Ru Zhao, Zhi-Yuan Yao

**Affiliations:** 1 Department of Orthopedics, The Quzhou Affiliated Hospital of Wenzhou Medical University (Quzhou People’s Hospital), 100 Minjiang Avenue, Kecheng District, Quzhou, 324000 Zhejiang China; 2 Department of Orthopedics, The First Affiliated Hospital of Zhejiang Chinese Medical University (Zhejiang Provincial Hospital of Chinese Medicine), 54 Youdian Road, Shangcheng District, Hangzhou, 310006 Zhejiang China

**Keywords:** Metagenomic next-generation sequencing, Tissue culture technique, Spinal infection, Medical research, Infectious diseases, Diagnosis, Infection

## Abstract

Spinal infections pose a significant clinical challenge due to the difficulty of early diagnosis. Traditional Tissue Culture Technique (TCT) has limitations, while Metagenomic Next-Generation Sequencing (mNGS) has emerged as a promising diagnostic tool. However, reports on the diagnostic performance of mNGS and TCT for spinal infections are inconsistent, and there has been a lack of systematic analysis of the evidence. This systematic review and meta-analysis included 10 studies involving a total of 770 patients to compare the diagnostic accuracy of mNGS and TCT. A comprehensive literature search was conducted using PubMed, Embase, Web of Science, Cochrane Library, and SinoMed to identify studies evaluating the diagnostic accuracy of mNGS and TCT for spinal infections. Data were analyzed using Review Manager 5.3 and Stata 16.0 to compute the sensitivity, specificity, and Area Under the Summary Receiver Operating Characteristic Curve (AUC). The meta-analysis revealed pooled estimates for mNGS with a sensitivity of 0.81 (95% CI, 0.74–0.87), specificity of 0.75 (95% CI, 0.48–0.91), and an AUC of 0.85 (95% CI, 0.82–0.88). In contrast, pooled estimates for TCT showed a sensitivity of 0.34 (95% CI, 0.27–0.43), specificity of 0.93 (95% CI, 0.79–0.98), and an AUC of 0.59 (95% CI, 0.55–0.63). While mNGS demonstrates higher sensitivity and overall diagnostic accuracy than TCT, its lower specificity suggests that it may be most effective when used alongside conventional methods to enhance diagnostic reliability in spinal infections.

**Supplementary Information:**

The online version contains supplementary material available at 10.1038/s41598-025-06759-3.

## Introduction

Spinal infection, defined as an infectious process involving the vertebral body, intervertebral disc, paraspinal soft tissue, or epidural space and confirmed by clinical presentation, imaging findings, and microbiological or histopathological evidence, presents significant diagnostic challenges due to its insidious onset, non-specific symptoms, and often inconclusive laboratory findings^1–6^. Timely and accurate identification of pathogens is essential for guiding appropriate antimicrobial therapy. Traditionally, diagnosis has relied on isolating pathogens from cultured spinal tissue or fluid samples; however, the low detection rates of conventional microbiological cultures pose substantial challenges for accurate diagnosis and targeted antimicrobial therapy^7,8^.

Metagenomic next-generation sequencing (mNGS) has emerged as a promising diagnostic method for infectious diseases due to its ability to detect a broad range of pathogens, including rare and unculturable organisms^9–13^. Compared to TCT, mNGS typically offers faster turnaround and higher detection rates. However, its diagnostic accuracy in spinal infections remains controversial, with studies reporting considerable variability in sensitivity and specificity.

For instance, Zhang Yi et al.^14^, in a prospective study involving 38 patients with suspected spinal infections, found that mNGS had a sensitivity of 0.86 and specificity of 1.00, while TCT showed a sensitivity of 0.49 and the same specificity of 1.00. In contrast, Ma Chi-yuan et al.^15^ reported lower diagnostic performance, with mNGS yielding a sensitivity of 0.69 and specificity of 0.27, and TCT showing even poorer sensitivity at 0.15 but a similarly high specificity of 1.00. These inconsistencies may be attributed to differences in sample sizes, specimen acquisition methods (e.g., open surgery versus percutaneous biopsy), and sequencing platforms used across studies. These variations highlight the need for a systematic evaluation to synthesize current evidence and identify the true diagnostic performance of mNGS compared to TCT.

To date, no meta-analysis has consolidated the diagnostic efficacy of mNGS and TCT in spinal infections. This study aims to assess the diagnostic value of both mNGS and TCT for spinal infections by including analyses from studies that utilized both testing methods.

## Materials and methods

### Search strategy

This study was registered in the PROSPERO database (CRD42022383002) and adhered to the Preferred Reporting Items for Systematic Reviews and Meta-Analyses (PRISMA) guidelines. The PubMed, Embase, web of science, Cochrane library and SinoMed databases were searched until December 2023, respectively. The literature on the comparative diagnostic accuracy of mNGS and TCT was searched using specific terms including “mNGS”, “metagenomic next-generation sequencing”, “culture”, “spinal”, “spine”, “infection”, “infective”, and “spinal infection”.

### Inclusion and exclusion criteria

Inclusion Criteria: (1) Studies involving subjects with suspected spinal infections; (2) Studies comparing the diagnostic performance of mNGS and TCT; (3) Studies containing necessary data for extraction, such as counts of False Positives (FP), True Positives (TP), False Negatives (FN), and True Negatives (TN); (4) Diagnostic criteria based on histopathological tests and the Infectious Diseases Society of America (IDSA) standards^16^. Exclusion Criteria: (1) Studies focusing solely on mNGS or TCT; (2) Studies lacking comparative analysis of mNGS and TCT; (3) Studies with incomplete data; (4) Case reports, animal studies, limited analyses, and review comments.

### Data extraction

Two independent researchers(YZY, YCL) initially screened titles and abstracts according to the inclusion and exclusion criteria, followed by full-text reviews of eligible studies. Discrepancies were resolved through discussion. Common reference standards used for diagnosing spinal infections included histopathological tests and IDSA criteria. Extracted data comprised: (1) the first author’s name, year of publication, and study type; (2) number of cases and distribution in the diagnostic 4-fold table (FP, TP, FN, TN); (3) type of mNGS sequencing platform; (4) sampling method; (5) diagnostic reference standard for spinal infections.

### Quality assessment

The Quality Assessment of Diagnostic Accuracy Studies (QUADAS-2) tool was used to evaluate the methodological quality of included studies across four domains: patient selection, index test, reference standard, and flow/timing. Two independent researchers performed the assessment, with discrepancies resolved through group consensus. Studies classified as high risk in any domain were retained in the primary analysis but subjected to sensitivity analysis by excluding them to assess their impact on pooled estimates. Additionally, meta-regression was performed to evaluate whether QUADAS-2 risk categories (low/unclear/high) explained heterogeneity in sensitivity or specificity. No studies were excluded solely based on QUADAS-2 scores unless critical methodological flaws (e.g., non-consecutive enrollment without justification, lack of blinding between index test and reference standard) rendered diagnostic accuracy data unreliable.

### Statistical analysis

Review Manager 5.3 and Stata 16.0 were used to calculate pooled sensitivity, specificity, and 95% confidence intervals (CI) using a random-effects model to account for anticipated heterogeneity across studies. Summary Receiver Operating Characteristic (SROC) curves and the Area Under the Curve (AUC) were generated. Heterogeneity was quantified using the Q-test and I² statistic, with I² ≥ 50% indicating substantial heterogeneity. When significant heterogeneity (I² ≥ 50% or *p* < 0.1) was detected, threshold effect analysis, sensitivity analysis (e.g., excluding studies with high QUADAS-2 risk scores), and meta-regression were performed to identify sources of variability. All statistical tests used a significance threshold of *p* < 0.05.

## Results

### Search results and quality assessment

**Fig. 1:**
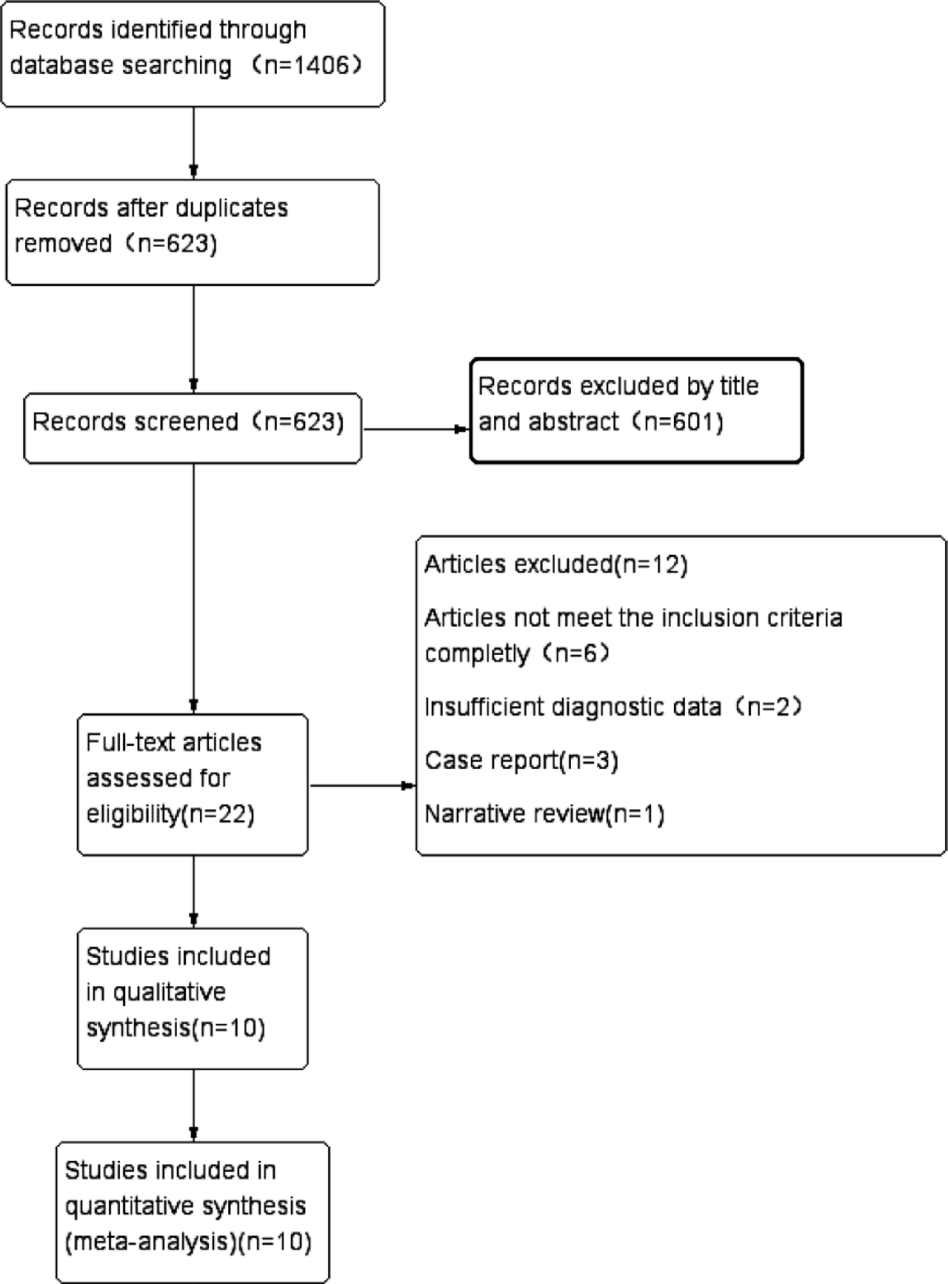
PRISMA flowchart of the study selection.

**Fig. 2:**
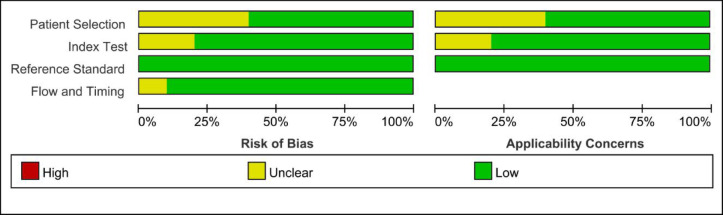
Risk of bias and applicability concerns graph.

**Fig. 3:**
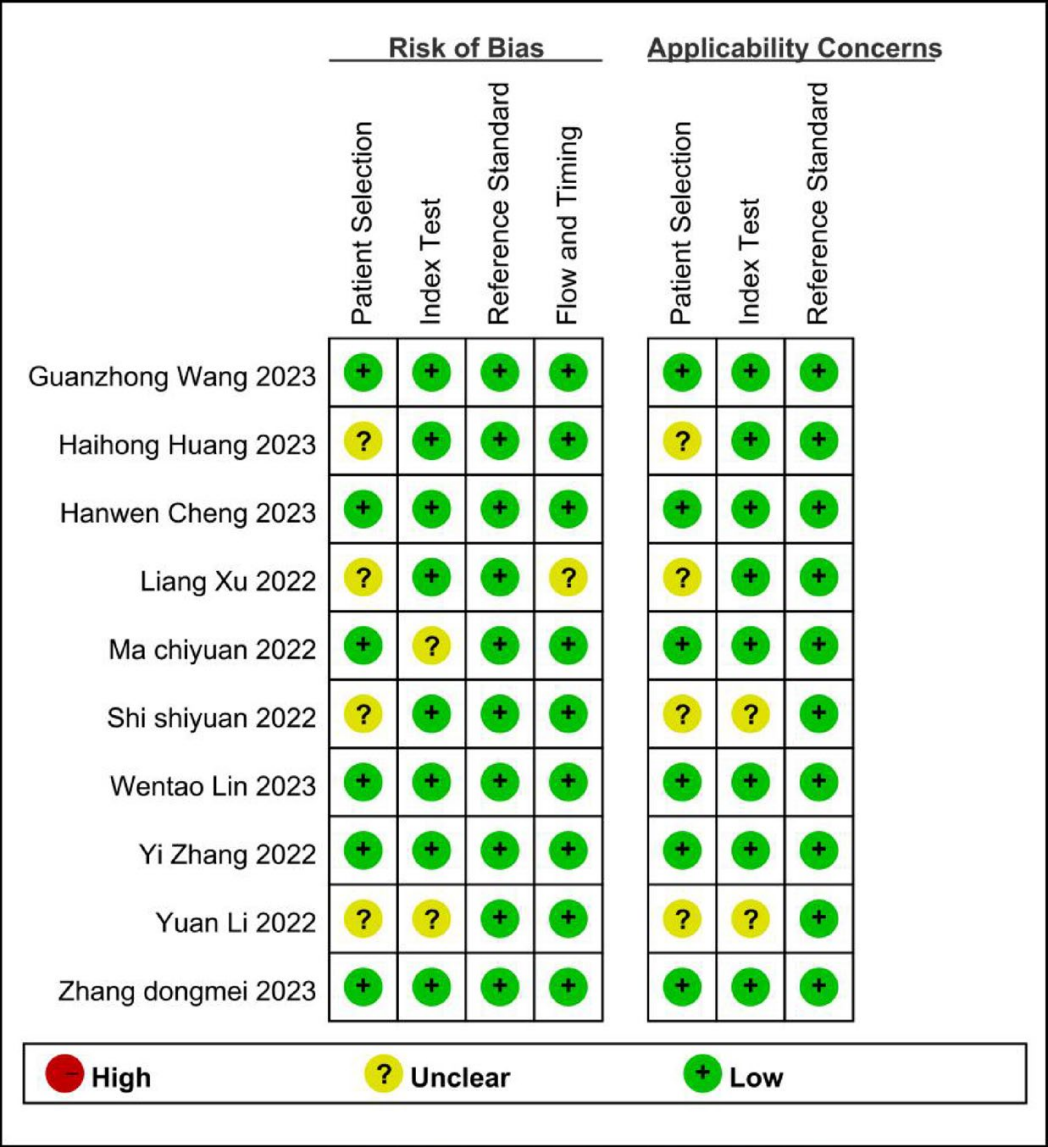
Risk of bias and applicability concerns summary.

The article selection process followed the PRISMA guidelines and is depicted in Fig. 1. A comprehensive search across the specified databases yielded 1,406 articles. After duplicate removal, 623 articles remained. Initial screening of titles and abstracts narrowed the selection to 22 articles. Full-text review led to the exclusion of 12 articles for various reasons: six did not meet the inclusion criteria, two lacked necessary data for extraction, three were case reports, and one was a narrative review. Ultimately, 10 studies were included in this review, consisting of two prospective and eight retrospective studies, involving a total of 770 patients. The basic characteristics of the included studies are summarized in Table 1. The methodological quality of these studies was assessed using the QUADAS-2 scale, as illustrated in Figs. 2 and 3. Most studies exhibited a low risk of bias concerning reference standards, procedures, and timing. However, four studies presented an unclear risk of bias regarding consecutive inclusion.

**Table 1:** Characteristics of the including studies.

Study	Study design	sample size	Cases (m: c)	TP m(c)	FP m(c)	FN m(c)	TN m(c)	Sampling methods	Sequencing Platform(mNGS)	Reference standards
Yi Zhang^14^	PS	38	38:37	30(17)	0(0)	5(18)	3(3)	PPS	Illumina NextSeq	Histopathology
Ma chiyuan^15^	RS	30	30:30	18(4)	8(0)	8(22)	3(4)	OS or PPS	MGISEQ	Histopathology
Haihong Huang^17^	RS	141	141:141	108(64)	2(1)	18(62)	13(14)	PPS	MGISEQ	Histopathology
Liang Xu^18^	RS	108	108:108	88(48)	2(7)	9(44)	9(9)	OS or PPS	Illumina NextSeq	IDSA
Wentao Lin^19^	RS	39	39:39	27(8)	1(1)	4(15)	7(7)	OS or PPS	MGISEQ	Histopathology
Hanwen Cheng^20^	RS	78	78:78	42(16)	1(1)	8(34)	27(27)	PPS	MGISEQ	Histopathology
Yuan Li^21^	PS	100	100:95	81(25)	8(0)	10(64)	1(6)	OS or PPS	MGISEQ	Histopathology
Guanzhong Wang^22^	RS	25	25:23	16(5)	0(0)	9(10)	4(3)	OS or PPS	MGISEQ	Histopathology
Shi shiyuan^23^	RS	171	171:171	76(43)	19(5)	60(93)	16(30)	OS or PPS	illumina Nextseq	IDSA
Zhang dongmei^24^	RS	40	40:40	23(5)	5(0)	5(23)	7(12)	OS or PPS	Illumina Nextseq	Histopathology

RS, Retrospective Study; PS, Prospective Study; m: c, mNGS group: tissue culture technique group; TP, true positive; FP, false positive; FN, false negative; TN, true negative; m(c), mNGS group(tissue culture technique group); IDSA, Infectious Diseases Society of America; PPS, Percutaneous puncture sampling; OS or PPS, open surgery or percutaneous puncture sampling.

### Meta-analysis results

#### Efficacy of the two testing methods

**Fig. 4:**
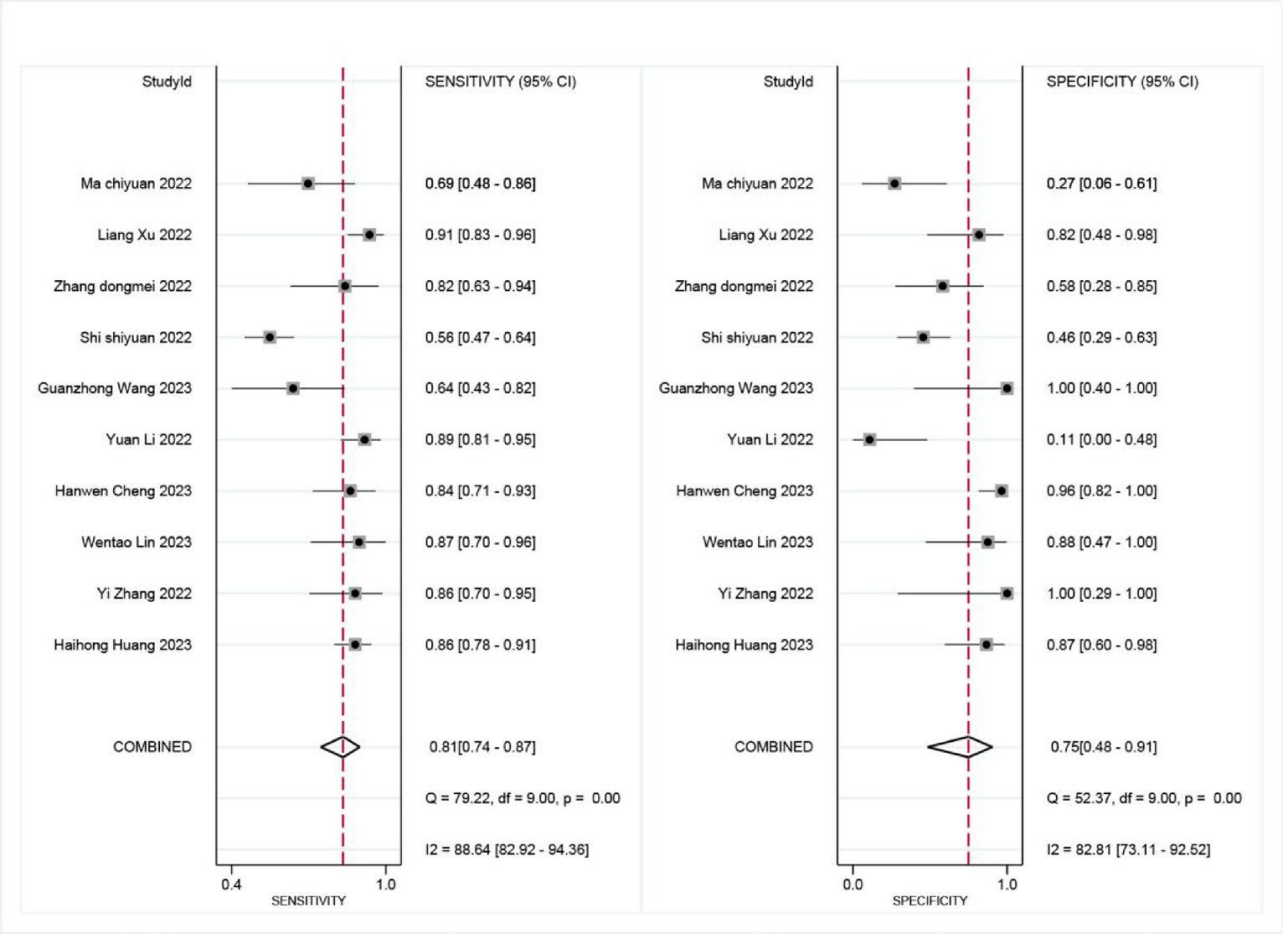
Sensitivity and specificity of mNGS for diagnosing spinal infections.

**Fig. 5:**
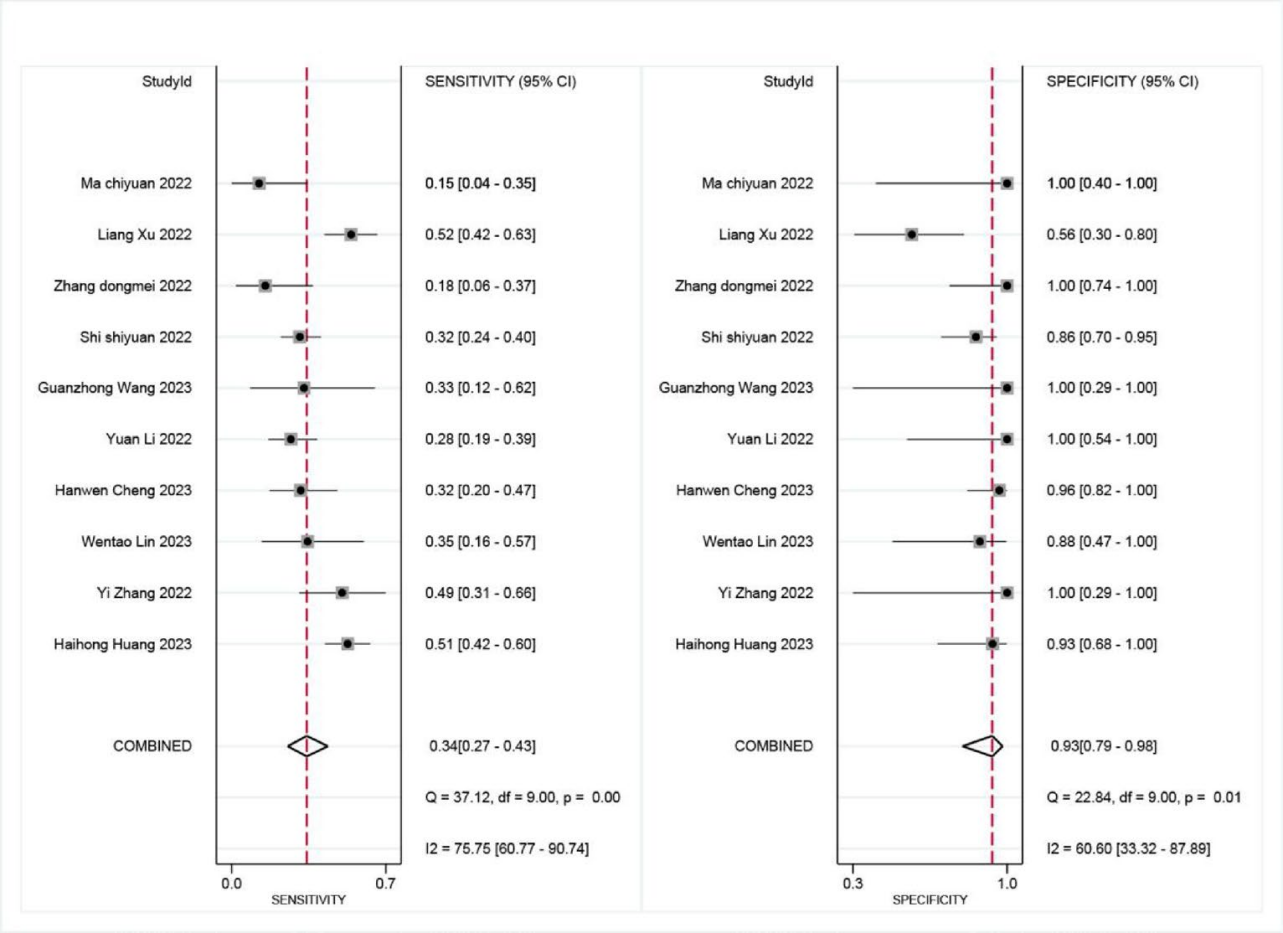
Sensitivity and specificity of TCT for diagnosing spinal infections.

**Fig. 6:**
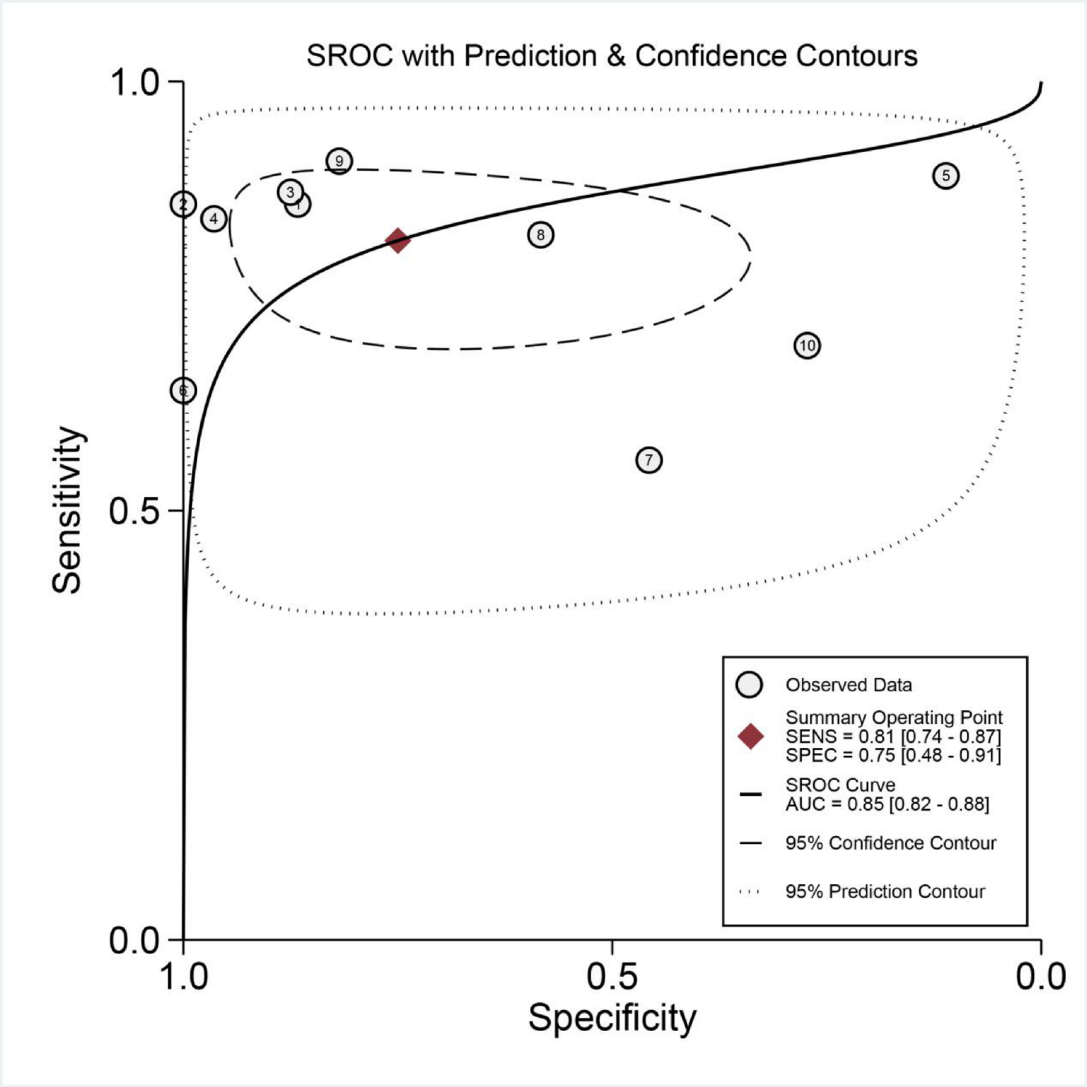
Summary receiver operator characteristic (SROC) curves based on mNGS.

**Fig. 7:**
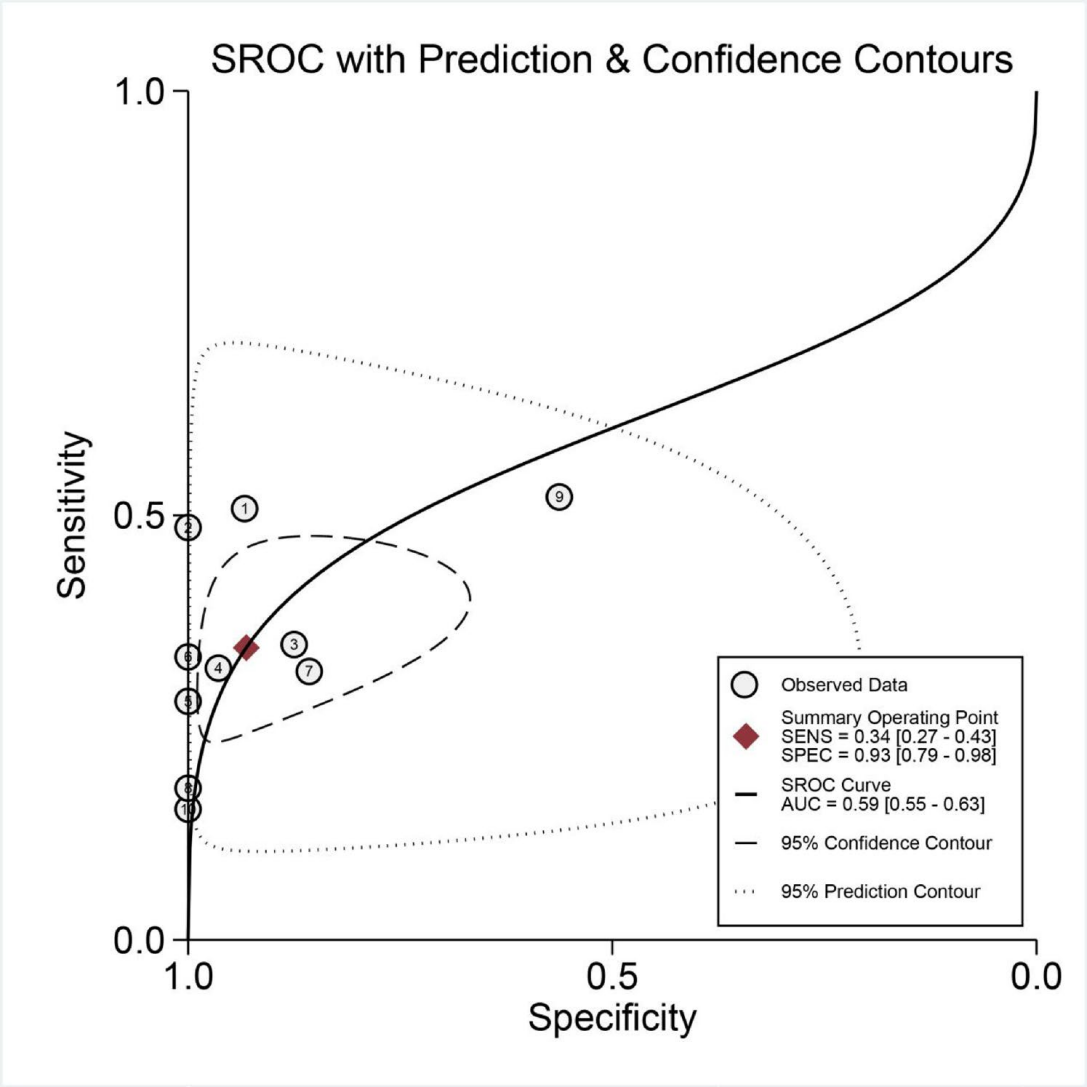
Summary receiver operator characteristic (SROC) curves based on TCT.

mNGS demonstrated a pooled sensitivity of 0.81 (95% CI, 0.74–0.87) and specificity of 0.75 (95% CI, 0.48–0.91) (Fig. 4). In contrast, TCT showed a pooled sensitivity of 0.34 (95% CI, 0.27–0.43) and specificity of 0.93 (95% CI, 0.79–0.98) (Fig. 5). The positive likelihood ratio (PLR) for mNGS was 3.30 (95% CI, 1.30–7.90) and for TCT was 5.00 (95% CI, 1.70–14.80), while the negative likelihood ratio (NLR) was 0.25 (95% CI, 0.15–0.41) for mNGS and 0.70 (95% CI, 0.63–0.79) for TCT. In clinical diagnostic interpretation, a PLR > 10 or NLR < 0.1 is generally considered to provide strong diagnostic evidence. Neither mNGS (PLR = 3.30, NLR = 0.25) nor TCT (PLR = 5.00, NLR = 0.70) met these criteria, demonstrating that neither method alone can definitively confirm or exclude spinal infection. The wide confidence intervals for mNGS specificity (0.48–0.91) and TCT sensitivity (0.27–0.43) further emphasize substantial variability across studies. These findings underscore the necessity of a combined diagnostic strategy integrating clinical evaluation, imaging, and microbiological testing to optimize diagnostic accuracy. To further evaluate diagnostic accuracy, SROC curves were plotted, revealing an AUC of 0.85 (95% CI, 0.82–0.88) for mNGS (Fig. 6) and 0.59 (95% CI, 0.55–0.63) for TCT (Fig. 7).

#### Heterogeneity analysis

**Fig. 8:**
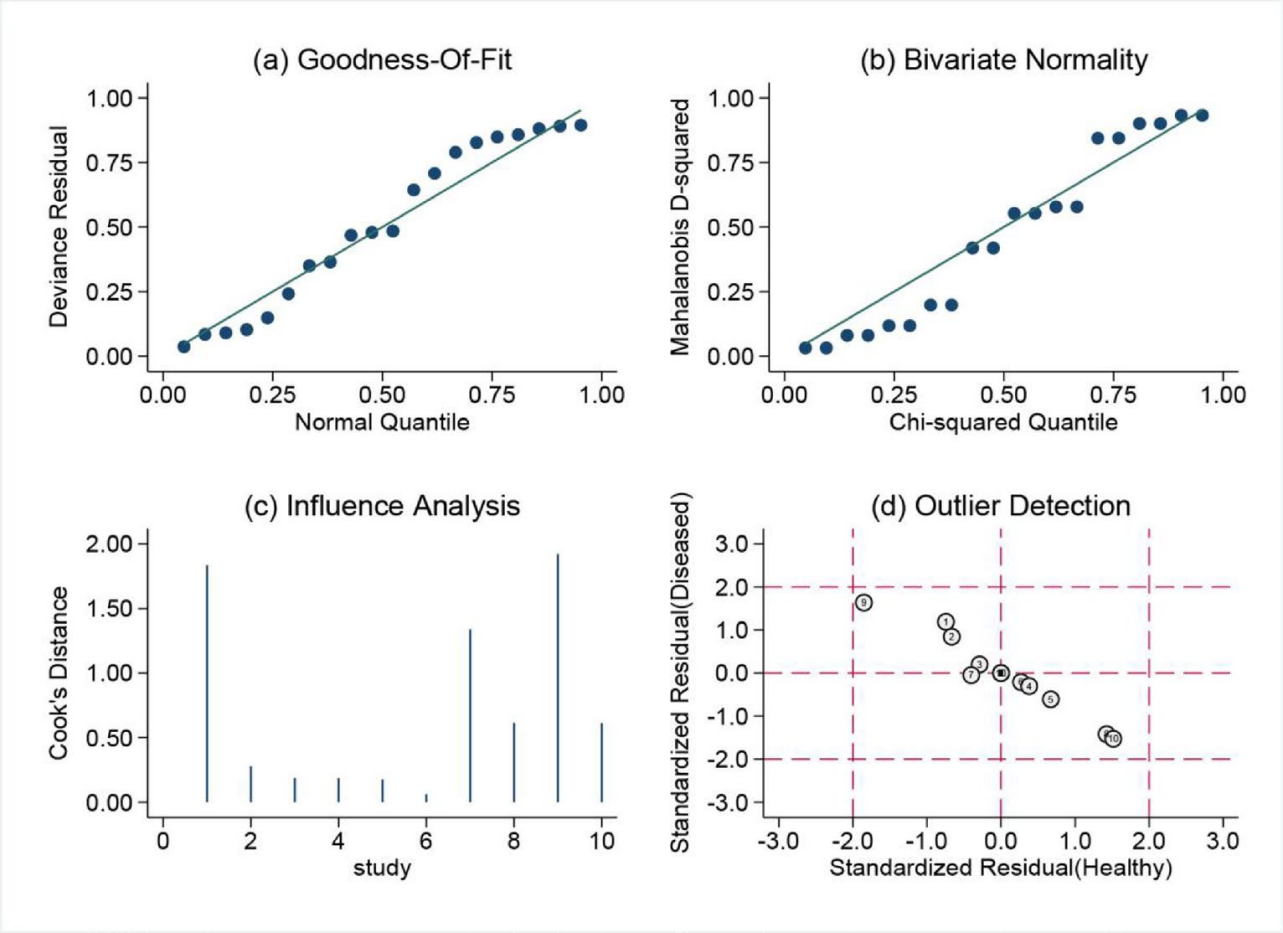
Sensitivity analysis of TCT group: Diagram of (**a**) Goodness-of-fit (**b**) Bivariate normality (**c**) Influence analysis (**d**) Outlier detection.

**Fig. 9:**
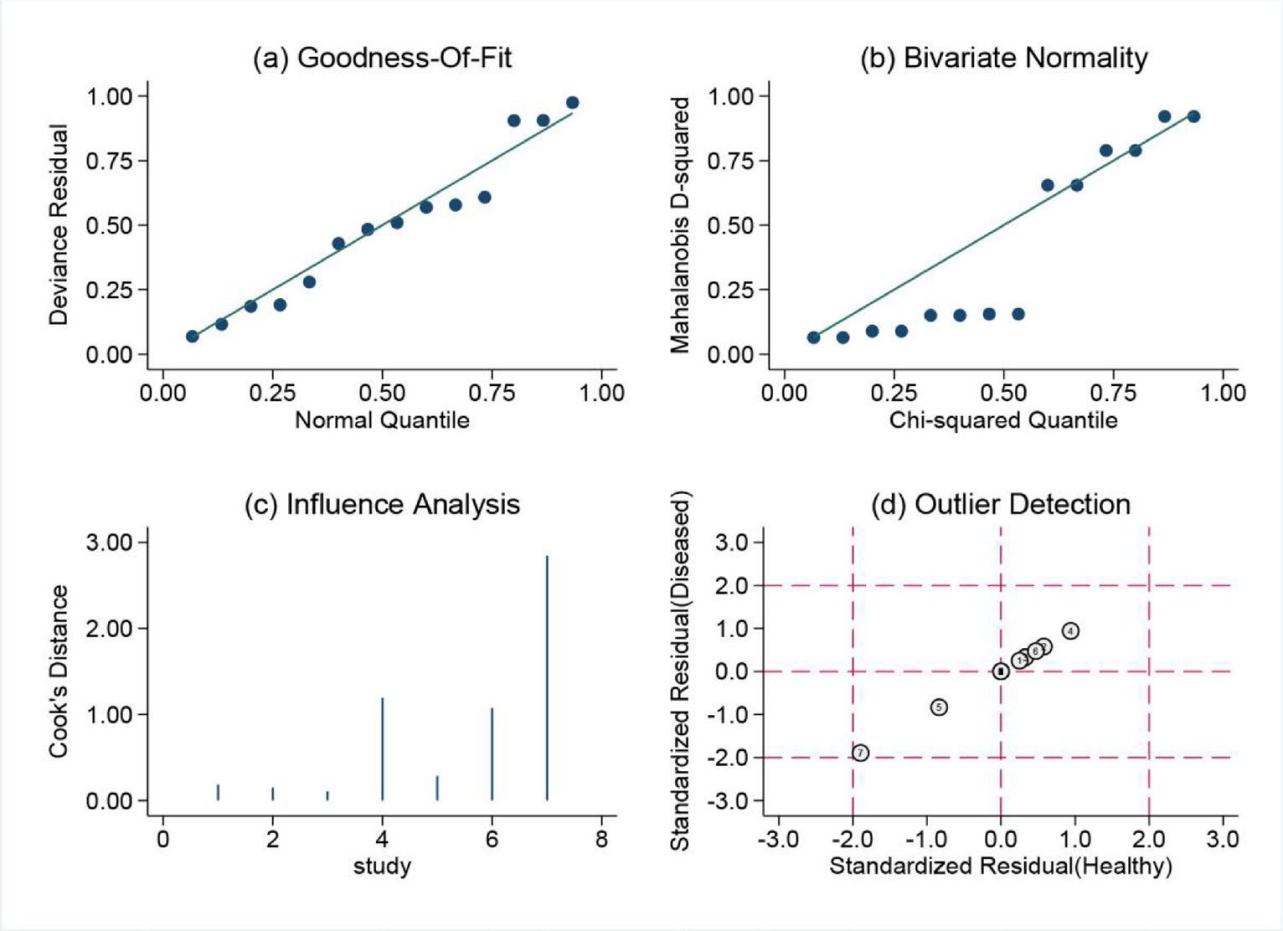
Sensitivity analysis of mNGS group after removing 3 studies: Diagram of (**a**) Goodness-of-fit (**b**) Bivariate normality (**c**) Influence analysis (**d**) Outlier detection.

Sensitivity analysis was conducted on the 10 studies included, assessing Goodness-of-fit, Bivariate normality, Influence analysis, and Outlier detection for the TCT group, as shown in Fig. 8. However, Influence analysis and Outlier detection identified studies by Yuan Li 2022^21^, Guanzhong Wang 2023^22^, and Shi Shiyuan 2022^23^ as potential sources of heterogeneity in the mNGS group (Fig. 9 and Supplementary Fig.S1). The Spearman correlation coefficient for sensitivity and specificity was 0.043 (*p* = 0.907) in the mNGS group and 0.411 (*p* = 0.238) in the TCT group, indicating that heterogeneity was not due to the threshold effect in either group. Further univariable meta-regression analysis was performed to explore the sources of heterogeneity. It was found that in the mNGS group, sensitivity was affected by sample size, and specificity was influenced by the sampling method. In the TCT group, sensitivity was influenced by the sampling method, while specificity was affected by both the sample size and the diagnostic reference standard (Tables 2 and 3, Supplementary Fig.S2 and S3).

**Table 2:** Meta-regression analysis of the mNGS group.

Variables	Subgroup	study	Sensitivity(95%CI)	*P*	Specificity(95%CI)	*P*
Sampling method	Percutaneous biopsy alone	3	0.86 (0.76–0.95)	0.32	0.95 (0.86–1.00)	0.01*
	Surgery or percutaneous biopsy	7	0.79 (0.71–0.88)		0.58 (0.35–0.80)	
Sample size	*n*<50	5	0.79 (0.68–0.90)	0.03^*^	0.79 (0.48–1.00)	0.63
	*n* ≥ 50	5	0.83 (0.75–0.91)		0.72 (0.42–1.00)	
Detection platform	Illumina NextSeq	4	0.80 (0.69–0.91)	0.06	0.74 (0.40–1.00)	0.95
	MGISEQ	6	0.82 (0.74–0.91)		0.76 (0.49–1.00)	
Reference standards	histopathology	8	0.83 (0.76–0.90)	0.06	0.67 (0.16–1.00)	0.84
	IDSA	2	0.76 (0.61–0.92)		0.77 (0.54–1.00)	

*Statistically significant if p-value < 0.05; IDSA, Infectious Diseases Society of America; CI, confidence interva.

**Table 3:** Meta-regression analysis of the TCT group.

Variables	Subgroup	Study	Sensitivity(95%CI)	*p*	Specificity(95%CI)	*p*
Sampling method	Percutaneous biopsy alone	3	0.44 (0.30–0.58)	0.03*	0.96 (0.88–1.00)	0.29
	Surgery or percutaneous biopsy	7	0.30 (0.22–0.39)		0.91 (0.81–1.00)	
Sample size	*n*<50	5	0.29 (0.18–0.41)	0.66	0.98 (0.92–1.00)	0.03*
	*n* ≥ 50	5	0.39 (0.29–0.49)		0.89 (0.77–1.00)	
Reference standards	histopathology	8	0.33 (0.24–0.42)	0.15	0.97 (0.93–1.00)	0.01*
	IDSA	2	0.41 (0.25–0.58)		0.74 (0.51–0.97)	

*Statistically significant if p-value < 0.05; IDSA, Infectious Diseases Society of America; CI, confidence interva.

Additionally, the Deeks funnel plot asymmetry test showed no significant publication bias in either the mNGS group (*p* = 0.59) or the TCT group (*p* = 0.96) (Supplementary Fig.S4 and S5).

In addition, meta-regression was performed to assess whether sequencing platform influenced diagnostic performance. No significant difference was observed in sensitivity (*p* = 0.06) or specificity (*p* = 0.95) between the Illumina and MGISEQ platforms.

## Discussion

In recent years, mNGS has been increasingly utilized for diagnosing spinal infections, achieving promising clinical outcomes. In contrast, TCT, a classical method in the diagnosis of spinal infections, often suffers from low culture-positive rates. Consensus on the relative efficacy of these two diagnostic methods is still lacking. Consequently, this study reviewed the existing literature with the hope of filling this gap and providing a foundation for evidence-based clinical decision-making.

Most studies have indicated that mNGS offers significant clinical value in diagnosing spinal infections^14,15,17^. Our findings demonstrate that mNGS is more sensitive than TCT (0.81 vs. 0.34), albeit with a lower specificity (0.75 vs. 0.93). The PLR for mNGS and TCT was 3.30 and 5.00, respectively, while the NLR was 0.25 for mNGS and 0.70 for TCT. It is widely recognized that a PLR > 10 or an NLR < 0.1 can decisively confirm or exclude a diagnosis. Our findings suggest that neither mNGS nor TCT alone can definitively confirm or rule out spinal infections. However, mNGS exhibited not only higher sensitivity but also a more favorable diagnostic efficacy and a higher AUC compared to TCT, underscoring its potential as a more effective diagnostic tool in clinical settings.

The studies included in this analysis exhibited considerable heterogeneity. Meta-regression results indicated that small sample sizes (*n* < 50) and sampling methods contributed to the heterogeneity observed in the mNGS groups, while for the TCT group, the same factors plus reference standards were influential. Specifically, five studies^14,15,19,22,24^ had sample sizes under 50, potentially affecting the pooled results for both groups. In terms of sampling techniques, percutaneous puncture was used exclusively in three studies, while a combination of open surgery and percutaneous puncture was employed in seven. Percutaneous puncture biopsy is a critical method for obtaining specimens in spinal infections^25^ and is noted for minimizing skin contamination at the puncture site^26^. Jakrapun et al.^27^ conducted a meta-analysis on the efficacy of image-guided percutaneous puncture vertebral biopsy in spinal infections, demonstrating that sampling methods significantly impact diagnostic outcomes. Auid-Orcid et al.^28^ investigated sampling methods from 31 patients with suspected spinal tuberculosis. They found that the sensitivity for diagnosing tuberculosis was significantly higher with open surgical biopsy compared to percutaneous Xpert testing. Similarly, the specificity also showed a significant difference, being higher in open surgical biopsy versus percutaneous Xpert testing. Jin et al.^29^ reported similar findings, where the sensitivity of Xpert for diagnosing tuberculosis varied between open surgery (0.84) and percutaneous puncture (0.77). Our findings indicate that the specificity of mNGS and the sensitivity of TCT could be influenced by the sampling methods used. Additionally, this study’s results suggest that the specificity of TCT is affected by the reference standards employed, which may contribute to TCT’s lower sensitivity and relatively poor detection capability.

Different detection platforms have varying criteria for microbial positivity, which can impact test outcomes^30,31^. Zhang et al.^32^ utilized both Illumina and Nanopore mNGS platforms to assess their ability to detect microorganisms in alveolar lavage fluid. Their findings indicated that the Nanopore platform was superior in detecting *Mycobacterium tuberculosis* and fungi. Li Yulian et al.^33^ conducted a meta-analysis on the use of mNGS to detect *Mycobacterium tuberculosis* in the lungs, highlighting that variations in detection platforms could contribute to the heterogeneity in sensitivity observed. The mNGS platforms discussed in this paper include Illumina NextSeq and MGISEQ. The Illumina NextSeq platform is noted for its higher gene coverage, while the MGISEQ platform boasts greater sensitivity^34^. Nevertheless, our analysis found that the choice of detection platform did not account for the heterogeneity observed within the mNGS group. Meta-regression analysis revealed no statistically significant difference in diagnostic performance between the Illumina and MGISEQ platforms (sensitivity: *p* = 0.06; specificity: *p* = 0.95). However, the small number of Illumina-based studies (*n* = 4) may have limited the statistical power to detect subtle differences. These findings are consistent with a previous meta-analysis by Sike et al.^35^, which evaluated mNGS for diagnosing cerebrospinal fluid infections in pediatric patients and found that diagnostic performance did not significantly vary across Illumina and BGISEQ platforms.

Although meta-regression was employed to investigate the sources of heterogeneity in each group, it could not fully account for the observed variability. This study has several limitations potentially linked to various factors: (1) Antibiotic exposure prior to sampling could influence outcomes, as pre-sampling antibiotic use or brief discontinuation might eradicate microorganisms in tissues, thus affecting detection rates in both groups^7,36,37^. Husseini et al.^38^ recommend suspending antibiotics two weeks before a puncture biopsy to optimize microbial yield; (2) The results might also be influenced by the location and timing of the sample collection. Anderson et al.^39^ found that pus from punctures yields a higher positivity rate than bone or disc tissues, and biopsies from the upper thoracic spine show higher positivity rates. Alessandro et al.^40^ investigated factors affecting tissue cultures and found no significant differences related to the volume of tissue extracted or the biopsy area (bone tissue vs. intervertebral discs); however, sampling during the acute phase notably increased microbial detection rates; (3) mNGS can theoretically detect all potential pathogens in a sample by extracting nucleic acids^41–43^, but the sequencing platform’s dataset length might impact the accuracy of detection and the estimation of abundance^44^. Interpretation of mNGS results could be skewed by the presence of underlying microbiota, sample contamination, and host nucleic acids^45^. Four studies in our analysis reported possible contamination-related false positives, accounting for approximately 5.6% of mNGS-positive samples (15 out of 266), primarily involving low-abundance or environmental organisms. Regrettably, most of the 10 studies included did not fully report such data, preventing a thorough analysis of the relevant heterogeneity. This reinforces the importance of interpreting sequencing results in conjunction with clinical findings, conventional microbiology, and imaging; (4) Due to the high sensitivity of mNGS, there is an inherent risk of false-positive results stemming from sample contamination, environmental microorganisms, or colonizing species, particularly in specimens with low microbial burden. This limitation underscores the need for cautious interpretation of mNGS results and highlights the importance of integrating molecular findings with clinical judgment, imaging studies, and conventional microbiology to enhance diagnostic accuracy; (5) In addition to methodological concerns, practical barriers such as cost and accessibility limit the clinical applicability of mNGS. The test is expensive, requires advanced sequencing platforms, and depends on trained personnel and bioinformatics infrastructure—resources that may not be readily available in many healthcare settings. These economic and logistical constraints should be considered when evaluating the feasibility of widespread implementation; (6) Although the study included dual-test data from the same patient cohorts, we did not apply a contrast-based or arm-based model due to limitations in cross-tabulated reporting and the complementary nature of our research objective. Future studies may explore these models in head-to-head comparisons with standardized paired data; (7) Due to inconsistent or missing pathogen-specific data across the included studies, we were unable to assess whether diagnostic performance varied by microorganism type. Future studies incorporating standardized microbiological classifications may help clarify test-specific strengths for different spinal pathogens.

A key strength of this study is that, to the best of our knowledge, it represents the first meta-analysis to systematically and directly compare the diagnostic performance of mNGS and TCT specifically in spinal infections. This study offers a distinct contribution to the current body of literature. By synthesizing data from ten studies across varied patient populations, sampling methods, and sequencing platforms, this analysis provides robust evidence to guide clinical practice.

## Conclusions

This study represents the first comparative meta-analysis of the diagnostic performance of mNGS versus TCT in spinal infections. The results demonstrate that mNGS provides higher sensitivity and overall diagnostic accuracy compared to TCT. However, its lower specificity indicates a risk of false positives, suggesting that mNGS may be more effective when used in conjunction with traditional diagnostic methods such as TCT to improve diagnostic confidence.

## Electronic supplementary material

Below is the link to the electronic supplementary material.

Supplementary Material 1

Supplementary Material 2

Supplementary Material 3

Supplementary Material 4

Supplementary Material 5

## Data Availability

The datasets are available from the corresponding author upon reasonable request.

## Abbreviations

mNGS: Metagenomic next generation sequencing
TCT: Tissue culture technique
AUC: Area under the summary receiver operating characteristic curve
PRISMA: Preferred reporting items for systematic reviews and meta-analyses
SROC: Summary receiver operating characteristic
CI: Confidence interval
FP: False positives
TP: True positives
FN: False negatives
TN: True negatives
IDSA: Infectious Diseases Society of America
PLR: The positive likelihood ratio
TN: The negative likelihood ratio
