# Supplementary material for: Metagenomic next-generation sequencing (mNGS) versus tissue culture technique (TCT) in diagnosis of spinal infection: a systematic review and meta-analysis
Source: Sci Rep. 2025 Jul 1;15:20926. doi: 10.1038/s41598-025-06759-3 (PMC12214740; doi:10.1038/s41598-025-06759-3)
Supplement: Supplementary file 1 — Supplementary Material 1 [file 41598_2025_6759_MOESM1_ESM.doc]

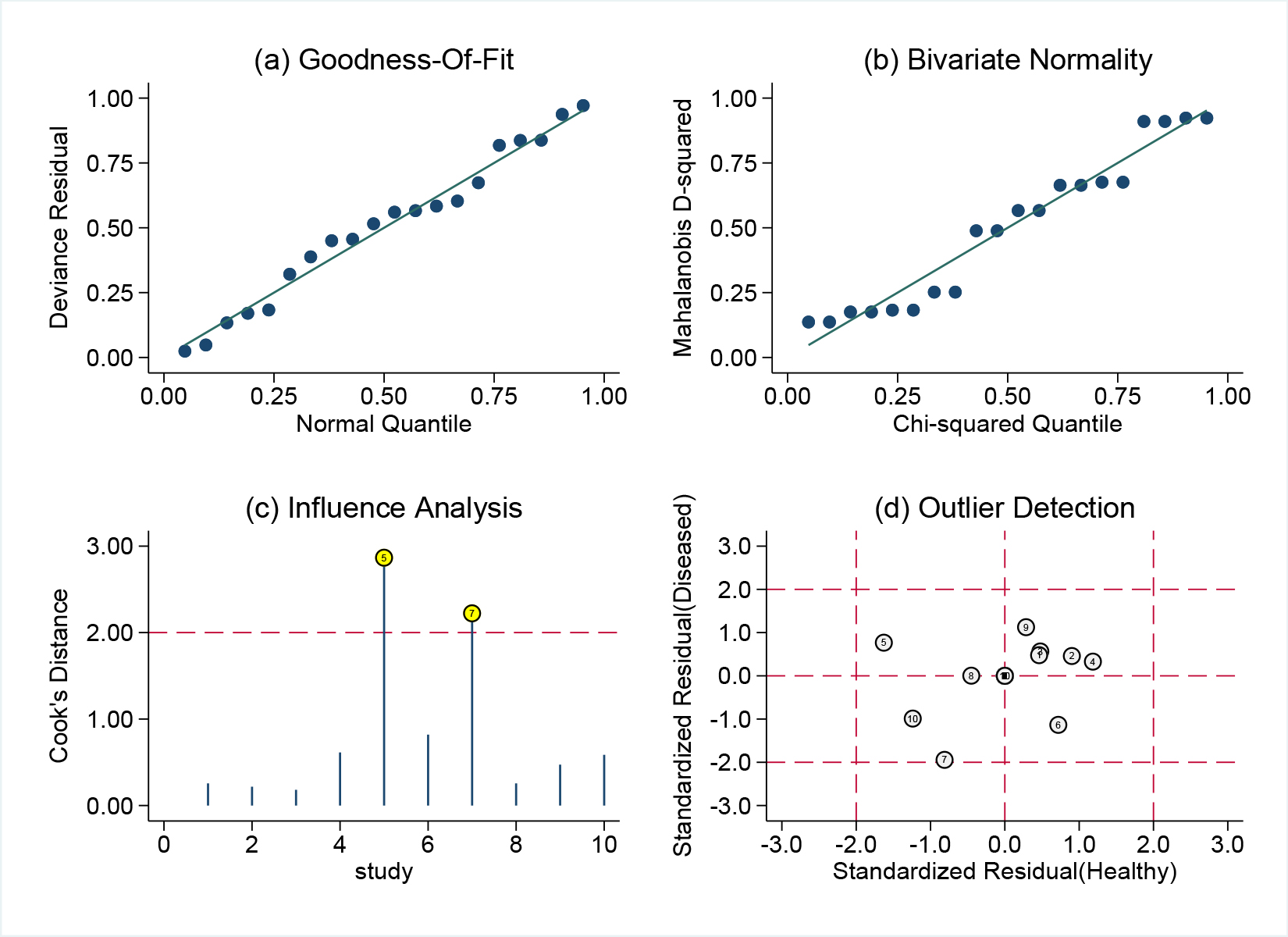


Supplementary Fig.S1 Sensitivity analysis indicated Yuan Li 2022[21], Guanzhong Wang 2023[22] and Shi Shiyuan 2022[23] might be the potential sources of heterogeneity in the mNGS group. (a)Goodness-of-fit；(b)Bivariate normality；(c) Influence analysis；(d)Outlier detection
