# Supplementary figures and images for: Metagenomic next-generation sequencing (mNGS) versus tissue culture technique (TCT) in diagnosis of spinal infection: a systematic review and meta-analysis
Source: Sci Rep. 2025 Jul 1;15:20926. doi: 10.1038/s41598-025-06759-3 (PMC12214740; doi:10.1038/s41598-025-06759-3)

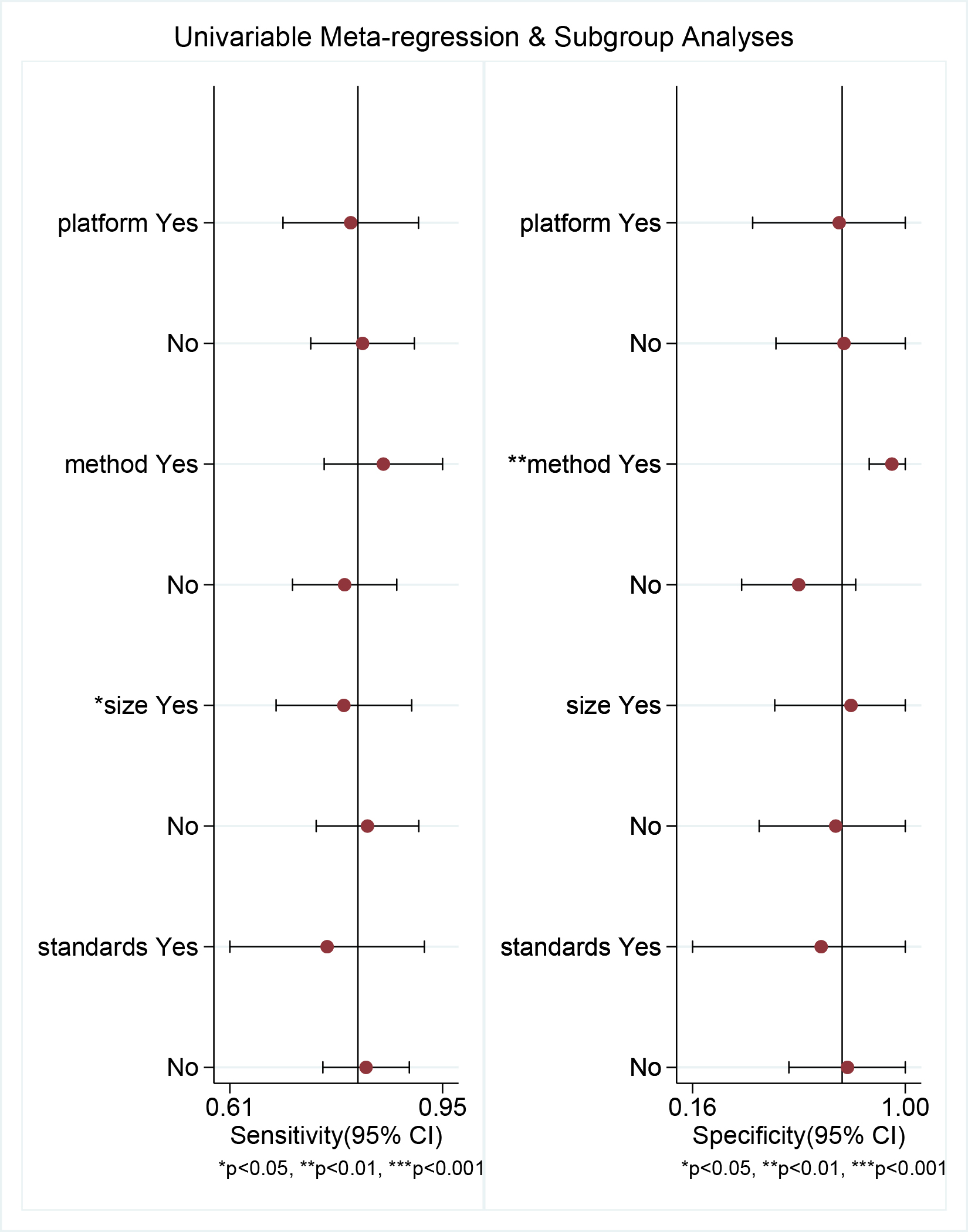

Supplement: Supplementary file 2 — Supplementary Material 2 [file 41598_2025_6759_MOESM2_ESM.jpg]

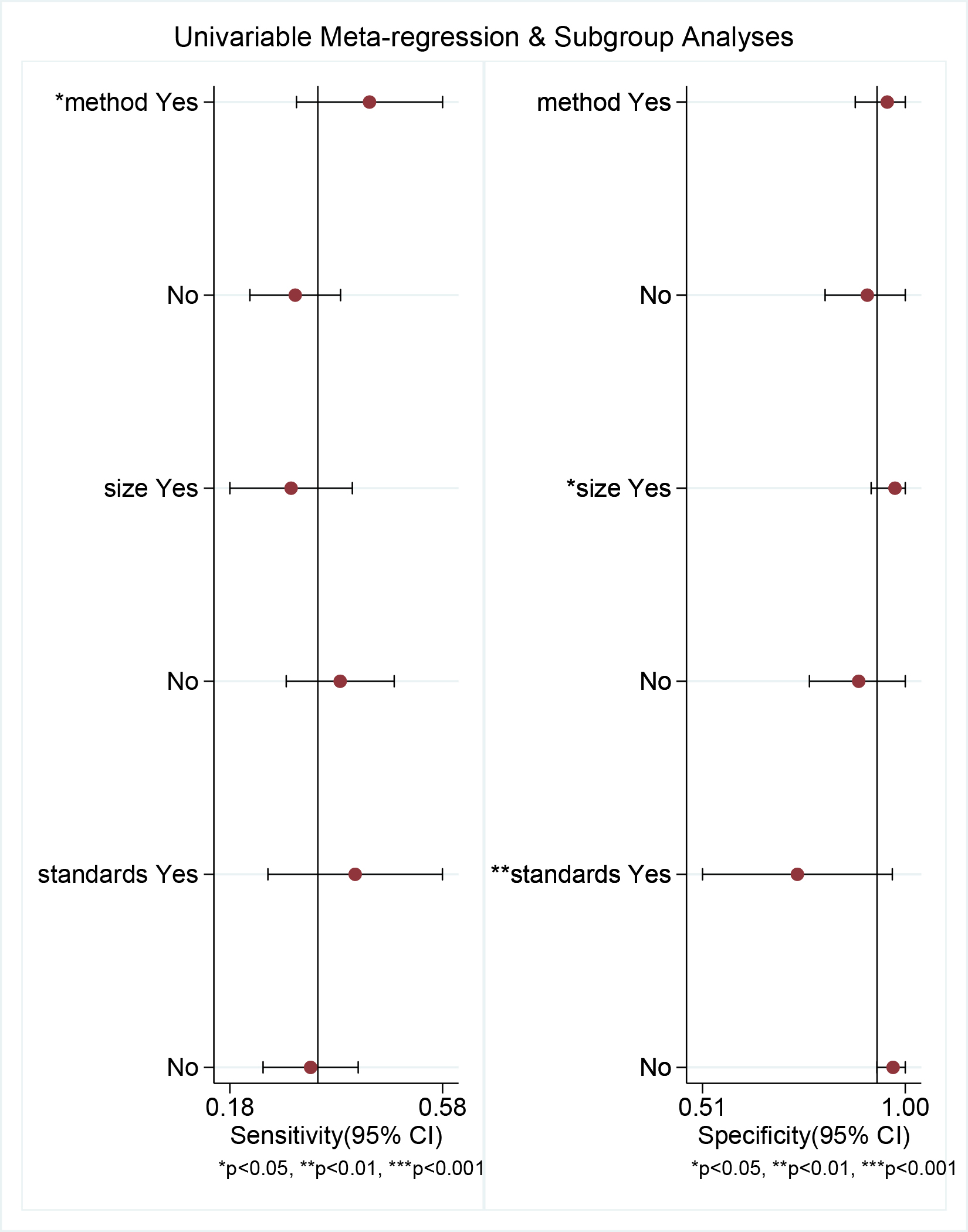

Supplement: Supplementary file 3 — Supplementary Material 3 [file 41598_2025_6759_MOESM3_ESM.jpg]

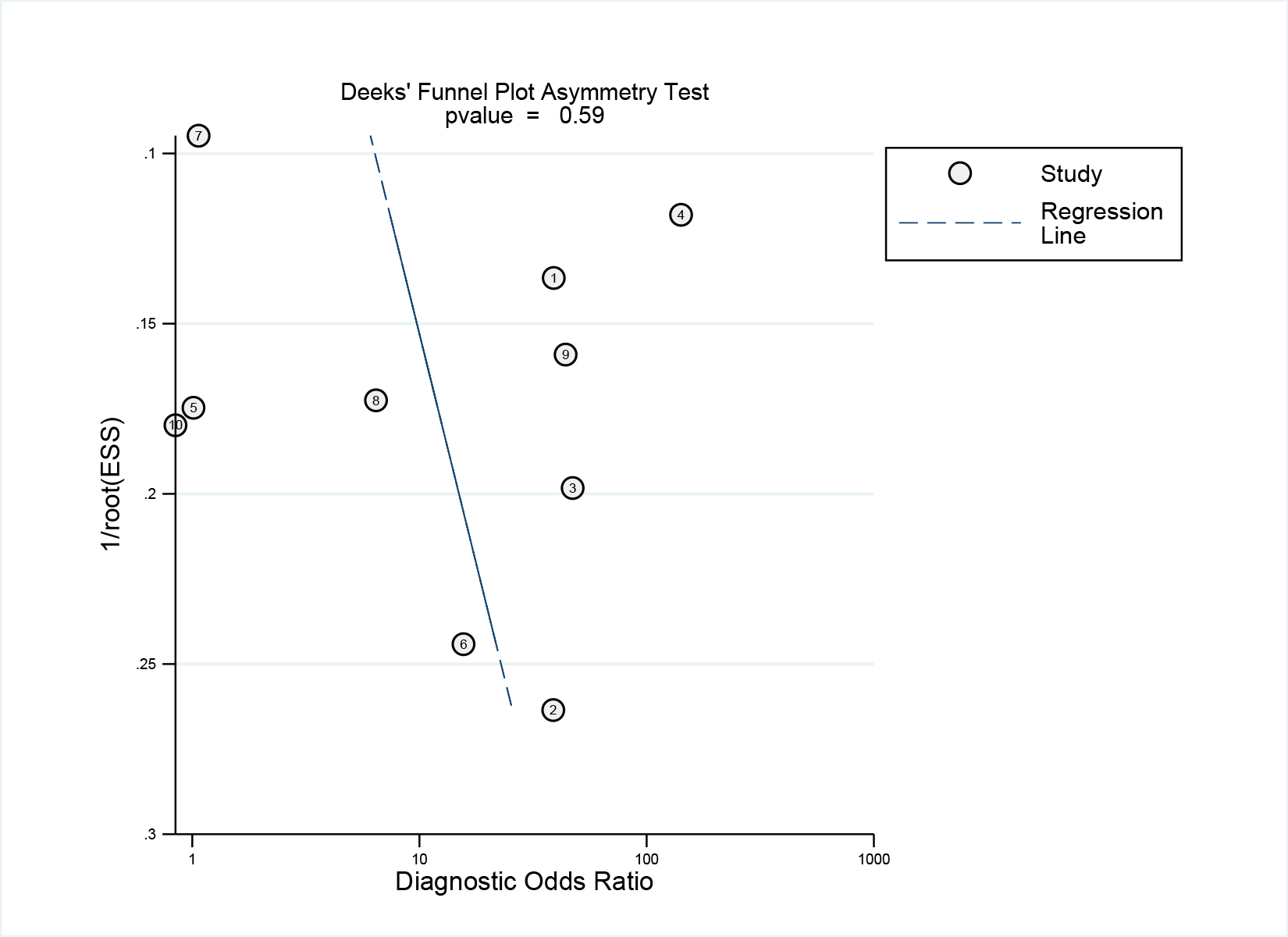

Supplement: Supplementary file 4 — Supplementary Material 4 [file 41598_2025_6759_MOESM4_ESM.jpg]

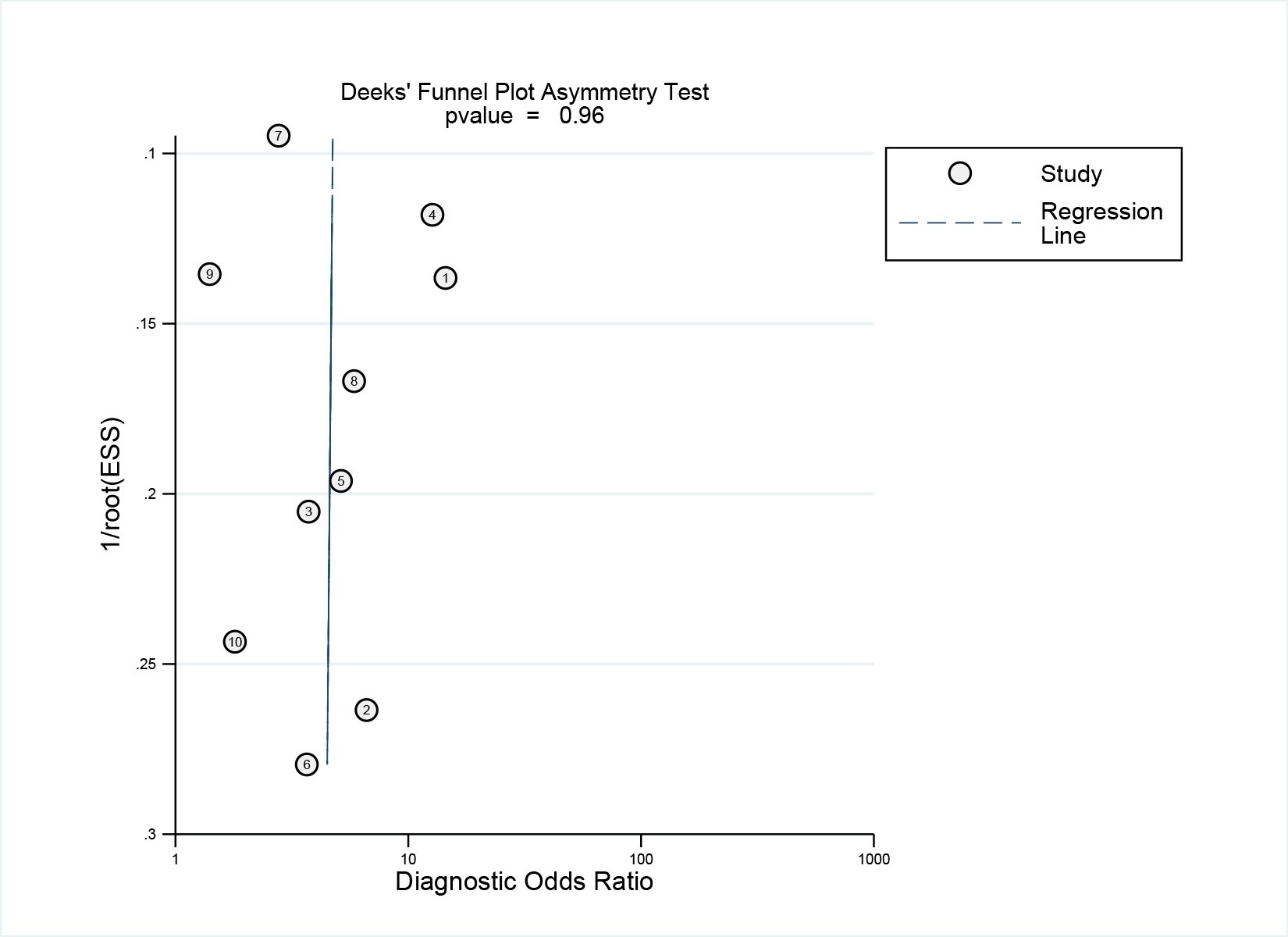

Supplement: Supplementary file 5 — Supplementary Material 5 [file 41598_2025_6759_MOESM5_ESM.jpg]
